# 4-(9-Anthr­yl)-1-phenylspiro­[azetidine-3,9′-xanthen]-2-one

**DOI:** 10.1107/S1600536809004255

**Published:** 2009-02-11

**Authors:** Ísmail Çelik, Mehmet Akkurt, Aliasghar Jarrahpour, Edris Ebrahimi, Orhan Büyükgüngör

**Affiliations:** a Department of Physics, Faculty of Arts and Sciences, Cumhuriyet University, 58140 Sivas, Turkey; b Department of Physics, Faculty of Arts and Sciences, Erciyes University, 38039 Kayseri, Turkey; c Department of Chemistry, College of Sciences, Shiraz University, 71454 Shiraz, Iran; d Department of Physics, Faculty of Arts and Sciences, Ondokuz Mayıs University, 55139 Samsun, Turkey

## Abstract

The β-lactam ring of the title compound, C_35_H_23_NO_2_, is nearly planar with a maximum deviation of 0.003 (3) Å from the mean plane. It makes dihedral angles of 17.4 (2), 85.22 (17) and 65.39 (16)°, respectively, with the phenyl, xanthene and anthracene ring systems. In the crystal structure, there are intra­molecular C—H⋯O and C—H⋯N contacts and mol­ecules are also linked by C—H⋯π inter­actions.

## Related literature

For general background on β-lactam anti­biotics, see: Banik *et al.* (2003 ▶); Jarrahpour & Khalili (2007 ▶); Miller (2000 ▶); Palomo *et al.* (2004 ▶). For the crystal structures of related compounds, see: Akkurt, Jarrahpour *et al.* (2008 ▶); Akkurt, Karaca *et al.* (2008 ▶); Pınar *et al.* (2006 ▶). For geometric analysis, see: Cremer & Pople (1975 ▶).
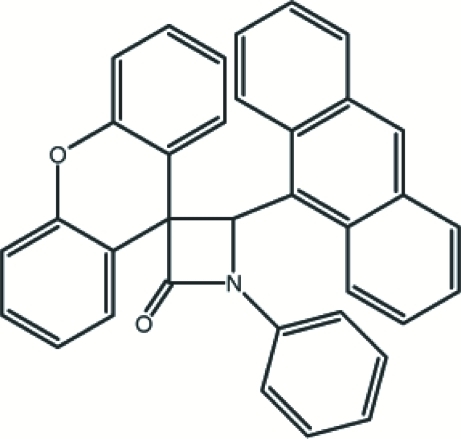


## Experimental

### 

#### Crystal data

C_35_H_23_NO_2_
*M* *_r_* = 489.54
Monoclinic, 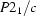

*a* = 13.6906 (8) Å
*b* = 13.3085 (7) Å
*c* = 17.3527 (10) Å
β = 127.548 (4)°
*V* = 2506.7 (3) Å^3^
*Z* = 4
Mo *K*α radiation
μ = 0.08 mm^−1^
*T* = 295 (2) K
0.24 × 0.18 × 0.14 mm

#### Data collection

STOE IPDS 2 diffractometer
Absorption correction: integration (**X-RED32**; Stoe & Cie, 2002 ▶) *T*_min_ = 0.981, *T*_max_ = 0.989
19631 measured reflections
5191 independent reflections
2442 reflections with *I* > 2σ(*I*)
*R*_int_ = 0.067

#### Refinement

*R*[*F* ^2^ > 2σ(*F* ^2^)] = 0.051
*wR*(*F* ^2^) = 0.110
*S* = 0.90
5191 reflections
343 parameters
H-atom parameters constrained
Δρ_max_ = 0.23 e Å^−3^
Δρ_min_ = −0.13 e Å^−3^

### 

Data collection: *X-AREA* (Stoe & Cie, 2002 ▶); cell refinement: *X-AREA*; data reduction: *X-RED32* (Stoe & Cie, 2002 ▶); program(s) used to solve structure: *SIR97* (Altomare *et al.*, 1999 ▶); program(s) used to refine structure: *SHELXL97* (Sheldrick, 2008 ▶); molecular graphics: *ORTEP-3* (Farrugia, 1997 ▶); software used to prepare material for publication: *WinGX* (Farrugia, 1999 ▶).

## Supplementary Material

Crystal structure: contains datablocks global, I. DOI: 10.1107/S1600536809004255/is2388sup1.cif

Structure factors: contains datablocks I. DOI: 10.1107/S1600536809004255/is2388Isup2.hkl

Additional supplementary materials:  crystallographic information; 3D view; checkCIF report

## Figures and Tables

**Table 1:** Hydrogen-bond geometry (Å, °)

*D*—H⋯*A*	*D*—H	H⋯*A*	*D*⋯*A*	*D*—H⋯*A*
C2—H2⋯N1	0.93	2.28	2.964 (4)	130
C31—H31⋯O2	0.93	2.48	3.092 (3)	124
C3—H3⋯*Cg*1^i^	0.93	2.86	3.601 (3)	138
C11—H11⋯*Cg*2^ii^	0.93	2.63	3.543 (3)	166

Symmetry codes: (i) 
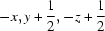
; (ii) 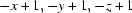. *Cg*1 and *Cg*2 are the centroids of the benzene ring (C8–C13) and phenyl ring (C30–C35), respectively.

**Fig. 1.**
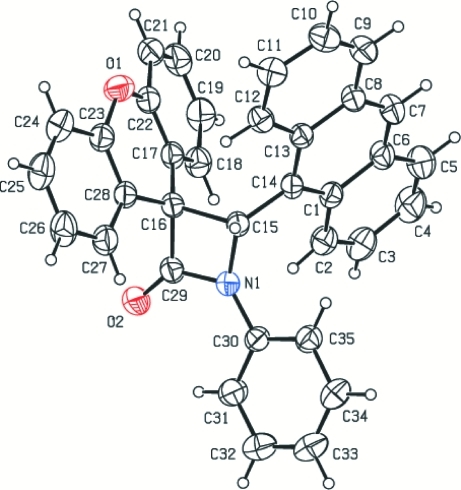
The title molecular structure, with the atom-numbering scheme and 30% probability displacement ellipsoids

**Fig. 2.**
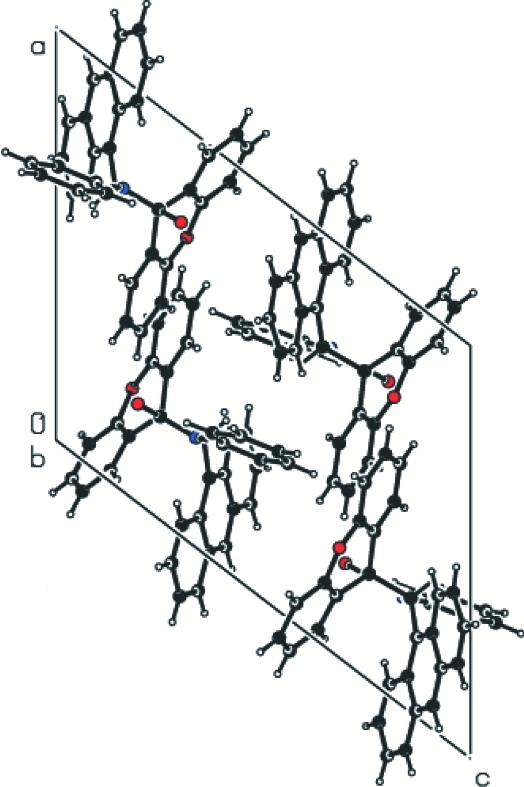
A view down the b axis of the packing of (I).

## supplementary crystallographic information

### Comment

The stereo selective synthesis of β-lactams has received considerable attention over recent years because of their wide variety of biological activities (Miller, 2000), in particular, asymmetric synthesis by means of a Staudinger ketene–imine reaction has been extensively studied (Palomo *et al.*, 2004). Several syntheses of spiro-β-lactams are available in the literature (Jarrahpour & Khalili, 2007; Pınar *et al.*, 2006; Akkurt, Jarrahpour *et al.*, 2008; Akkurt, Karaca *et al.*, 2008). The synthesis of polycyclic aromatic β-lactams from polyaromatic imines have been reported in literature (Banik *et al.*, 2003).

The β-lactam unit in (I) (Fig. 1) is essentially planar, with a maximum deviation of 0.003 (3) Å from the mean plane. The β-lactam ring makes a dihedral angle of 17.4 (2)° with the phenyl ring C30—C35. In the xanthene ring system, attached at C16, the benzene rings (C17–C22) and (C23–C28) are almost planar, the dihedral angle between them 17.20 (14)°. Its central ring, C16/C17/C22/O1/C23/C28, is not planar, with puckering parameters: Q_T_ = 0.247 (3) Å, θ = 98.9 (7)° and φ = 355.1 (7)° (Cremer & Pople, 1975). The mean plane of the xanthene ring system forms the dihedral angles of 85.22 (17) and 85.62 (13)°, with the β-lactam ring and the phenyl ring, respectively. The anthracene ring system, attached at C15, is almost planar, with maximum deviations of 0.041 (2) Å for C1, -0.049 (3) Å for C4 and, -0.067 (2) Å for C13, makes dihedral angle of 65.39 (16), 80.60 (13) and 56.63 (8)°, with the β-lactam, the phenyl and the mean plane of the xanthene ring system, respectively.

In the crystal structure, there are intramolecular C—H···O and C—H···N contacts and molecules are linked to each other by C—H···π interactions between the adjacent molecules (Table 1 and Fig. 2) [*Cg*1 and *Cg*2 are the centroids of the benzene ring (C8–C13) and phenyl ring (C30–C35), respectively].

### Experimental

A mixture of (*E*)—*N*-(antheracen-9-ylmethylene)aniline (0.30 g, 1.07 mmol) and triethylamine (0.73 g, 7.21 mmol), 9*H*-xanthen-9-carboxylic acid (0.49 g, 2.17 mmol) and tosyl chloride (0.42 g, 2.20 mmol) in CH_2_Cl_2_ (15 ml) was stirred at room temperature for 24 h. Then it was washed with HCl 1 N (20 ml) and saturated sodiumbicarbonate solution (20 ml), brine (20 ml), dried (Na_2_SO_4_) and the solvent was evaporated to give the crude product as a light yellow crystal which was then purified by recrystallization from ethyl acetate (Yield 63%). dec: 511–513 K. IR (KBr, cm^-1^): 1758 (CO β-lactam). ^1^H-NMR δ (p.p.m.): 6.34 (s, 1H, 4), 6.51–8.83 (m, ArH, 22H).^13^C-NMR δ (p.p.m.): 65.6 (C-3), 75.4 (C-4), 115.9–152.0 (aromatic carbon), 167.5 (CO β-lactam). Analysis calculated for C_35_H_23_NO_2_: C 85.87, H 4.74, N 2.86%. Found: C 85.87, H 4.74, N 2.86%.

### Refinement

The H atoms were positioned geometrically and refined a riding model, with the C—H = 0.93 and 0.98 Å and with *U*_iso_(H) = 1.2*U*_eq_(C).

### Crystal data

**Table:**

C_35_H_23_NO_2_	*F*(000) = 1024
*M**_r_* = 489.54	*D*_x_ = 1.297 Mg m^−^^3^
Monoclinic, *P*2_1_/*c*	Mo *K*α radiation, λ = 0.71073 Å
Hall symbol: -P 2ybc	Cell parameters from 12888 reflections
*a* = 13.6906 (8) Å	θ = 1.5–28.1°
*b* = 13.3085 (7) Å	µ = 0.08 mm^−^^1^
*c* = 17.3527 (10) Å	*T* = 295 K
β = 127.548 (4)°	Block, colourless
*V* = 2506.7 (3) Å^3^	0.24 × 0.18 × 0.14 mm
*Z* = 4	

### Data collection

**Table:**

STOE IPDS 2 diffractometer	5191 independent reflections
Radiation source: sealed X-ray tube, 12 x 0.4 mm long-fine focus	2442 reflections with *I* > 2σ(*I*)
plane graphite	*R*_int_ = 0.067
Detector resolution: 6.67 pixels mm^-1^	θ_max_ = 26.5°, θ_min_ = 1.9°
ω scans	*h* = −17→17
Absorption correction: integration (*X-RED32*; Stoe & Cie, 2002)	*k* = −16→16
*T*_min_ = 0.981, *T*_max_ = 0.989	*l* = −21→21
19631 measured reflections	

### Refinement

**Table:**

Refinement on *F*^2^	Primary atom site location: structure-invariant direct methods
Least-squares matrix: full	Secondary atom site location: difference Fourier map
*R*[*F*^2^ > 2σ(*F*^2^)] = 0.051	Hydrogen site location: inferred from neighbouring sites
*wR*(*F*^2^) = 0.110	H-atom parameters constrained
*S* = 0.90	*w* = 1/[σ^2^(*F*_o_^2^) + (0.0406*P*)^2^] where *P* = (*F*_o_^2^ + 2*F*_c_^2^)/3
5191 reflections	(Δ/σ)_max_ < 0.001
343 parameters	Δρ_max_ = 0.23 e Å^−^^3^
0 restraints	Δρ_min_ = −0.13 e Å^−^^3^

### Special details

**Table:**

Geometry. Bond distances, angles *etc*. have been calculated using the rounded fractional coordinates. All su's are estimated from the variances of the (full) variance-covariance matrix. The cell e.s.d.'s are taken into account in the estimation of distances, angles and torsion angles
Refinement. Refinement on *F*^2^ for ALL reflections except those flagged by the user for potential systematic errors. Weighted *R*-factors *wR* and all goodnesses of fit *S* are based on *F*^2^, conventional *R*-factors *R* are based on *F*, with *F* set to zero for negative *F*^2^. The observed criterion of *F*^2^ > σ(*F*^2^) is used only for calculating -*R*-factor-obs *etc*. and is not relevant to the choice of reflections for refinement. *R*-factors based on *F*^2^ are statistically about twice as large as those based on *F*, and *R*-factors based on ALL data will be even larger.

### Fractional atomic coordinates and isotropic or equivalent isotropic displacement parameters (Å^2^)

**Table:**

	*x*	*y*	*z*	*U*_iso_*/*U*_eq_	
O1	0.26666 (17)	0.35613 (13)	0.18443 (13)	0.0741 (7)	
O2	0.23966 (18)	0.72931 (13)	0.19798 (13)	0.0792 (7)	
N1	0.26629 (17)	0.67174 (14)	0.33789 (13)	0.0537 (7)	
C1	0.0819 (2)	0.54484 (18)	0.34789 (15)	0.0537 (8)	
C2	0.0287 (2)	0.6401 (2)	0.30421 (17)	0.0654 (9)	
C3	−0.0826 (3)	0.6684 (2)	0.27919 (19)	0.0782 (11)	
C4	−0.1483 (3)	0.6065 (3)	0.2989 (2)	0.0925 (13)	
C5	−0.1006 (3)	0.5181 (3)	0.3433 (2)	0.0852 (11)	
C6	0.0134 (2)	0.4829 (2)	0.36802 (18)	0.0630 (9)	
C7	0.0576 (2)	0.3892 (2)	0.40911 (19)	0.0727 (11)	
C8	0.1641 (2)	0.34938 (19)	0.42885 (16)	0.0587 (9)	
C9	0.2069 (3)	0.2516 (2)	0.46931 (19)	0.0748 (11)	
C10	0.3073 (3)	0.2113 (2)	0.4844 (2)	0.0771 (11)	
C11	0.3703 (2)	0.2655 (2)	0.45823 (18)	0.0697 (10)	
C12	0.3355 (2)	0.35957 (19)	0.42191 (16)	0.0577 (9)	
C13	0.23202 (19)	0.40766 (18)	0.40696 (15)	0.0509 (8)	
C14	0.19341 (19)	0.50727 (18)	0.37026 (14)	0.0495 (8)	
C15	0.2778 (2)	0.56287 (16)	0.35636 (15)	0.0500 (8)	
C16	0.2588 (2)	0.54946 (17)	0.25569 (15)	0.0521 (8)	
C17	0.1450 (2)	0.49372 (19)	0.17648 (15)	0.0547 (8)	
C18	0.0275 (2)	0.5320 (2)	0.13170 (17)	0.0684 (10)	
C19	−0.0758 (2)	0.4769 (3)	0.06386 (19)	0.0788 (13)	
C20	−0.0634 (3)	0.3830 (3)	0.03877 (19)	0.0809 (13)	
C21	0.0515 (3)	0.3438 (2)	0.08025 (18)	0.0739 (11)	
C22	0.1546 (2)	0.3999 (2)	0.14835 (17)	0.0590 (9)	
C23	0.3694 (2)	0.4171 (2)	0.23285 (17)	0.0615 (9)	
C24	0.4717 (3)	0.3782 (2)	0.24527 (19)	0.0751 (11)	
C25	0.5788 (3)	0.4324 (3)	0.2946 (2)	0.0833 (13)	
C26	0.5831 (3)	0.5262 (3)	0.3300 (2)	0.0841 (13)	
C27	0.4806 (2)	0.5649 (2)	0.31657 (18)	0.0710 (10)	
C28	0.3711 (2)	0.51099 (19)	0.26791 (16)	0.0556 (9)	
C29	0.2507 (2)	0.66467 (19)	0.25206 (17)	0.0577 (9)	
C30	0.3017 (2)	0.75205 (18)	0.40329 (16)	0.0544 (8)	
C31	0.3090 (2)	0.8482 (2)	0.3781 (2)	0.0703 (10)	
C32	0.3526 (3)	0.9250 (2)	0.4456 (2)	0.0847 (11)	
C33	0.3860 (2)	0.9052 (2)	0.5366 (2)	0.0820 (11)	
C34	0.3757 (2)	0.8104 (2)	0.56045 (19)	0.0736 (10)	
C35	0.3336 (2)	0.7321 (2)	0.49401 (17)	0.0630 (9)	
H2	0.07130	0.68380	0.29260	0.0780*	
H3	−0.11600	0.72990	0.24850	0.0940*	
H4	−0.22450	0.62690	0.28120	0.1110*	
H5	−0.14300	0.47870	0.35840	0.1020*	
H7	0.01420	0.35130	0.42410	0.0870*	
H9	0.16430	0.21470	0.48570	0.0900*	
H10	0.33450	0.14780	0.51200	0.0920*	
H11	0.43750	0.23640	0.46600	0.0840*	
H12	0.38030	0.39400	0.40620	0.0690*	
H15	0.36340	0.54710	0.41060	0.0600*	
H18	0.01840	0.59610	0.14790	0.0820*	
H19	−0.15380	0.50350	0.03510	0.0950*	
H20	−0.13320	0.34550	−0.00660	0.0970*	
H21	0.05990	0.28020	0.06270	0.0890*	
H24	0.46790	0.31520	0.22020	0.0900*	
H25	0.64830	0.40610	0.30420	0.1000*	
H26	0.65550	0.56350	0.36310	0.1010*	
H27	0.48460	0.62850	0.34060	0.0850*	
H31	0.28480	0.86160	0.31600	0.0840*	
H32	0.35930	0.98990	0.42940	0.1020*	
H33	0.41570	0.95670	0.58200	0.0980*	
H34	0.39700	0.79780	0.62170	0.0880*	
H35	0.32710	0.66740	0.51050	0.0760*	

### Atomic displacement parameters (Å^2^)

**Table:**

	*U*^11^	*U*^22^	*U*^33^	*U*^12^	*U*^13^	*U*^23^
O1	0.0753 (12)	0.0664 (12)	0.0832 (12)	−0.0113 (10)	0.0496 (10)	−0.0175 (10)
O2	0.1112 (14)	0.0678 (12)	0.0720 (11)	−0.0093 (11)	0.0628 (11)	0.0030 (10)
N1	0.0632 (12)	0.0525 (13)	0.0528 (11)	−0.0055 (10)	0.0392 (10)	−0.0030 (9)
C1	0.0527 (13)	0.0623 (16)	0.0490 (13)	−0.0030 (12)	0.0325 (11)	−0.0062 (11)
C2	0.0601 (15)	0.0730 (19)	0.0648 (15)	0.0042 (14)	0.0390 (13)	−0.0026 (14)
C3	0.0683 (18)	0.091 (2)	0.0712 (17)	0.0159 (16)	0.0404 (15)	−0.0006 (15)
C4	0.0645 (18)	0.115 (3)	0.109 (2)	0.009 (2)	0.0585 (19)	−0.008 (2)
C5	0.0673 (18)	0.097 (2)	0.114 (2)	−0.0050 (17)	0.0669 (18)	−0.009 (2)
C6	0.0554 (14)	0.0742 (19)	0.0702 (15)	−0.0046 (13)	0.0438 (13)	−0.0050 (14)
C7	0.0760 (18)	0.080 (2)	0.0831 (18)	−0.0110 (16)	0.0593 (16)	−0.0021 (16)
C8	0.0619 (15)	0.0625 (17)	0.0603 (15)	−0.0054 (13)	0.0417 (13)	−0.0004 (12)
C9	0.090 (2)	0.0675 (19)	0.0786 (18)	−0.0088 (16)	0.0574 (17)	0.0034 (15)
C10	0.0818 (19)	0.0644 (18)	0.0797 (19)	0.0027 (16)	0.0465 (17)	0.0099 (14)
C11	0.0612 (16)	0.0695 (19)	0.0721 (17)	0.0044 (14)	0.0373 (14)	0.0034 (15)
C12	0.0494 (13)	0.0633 (17)	0.0566 (14)	−0.0037 (12)	0.0303 (12)	0.0012 (12)
C13	0.0504 (13)	0.0579 (15)	0.0438 (12)	−0.0076 (11)	0.0284 (11)	−0.0050 (10)
C14	0.0453 (12)	0.0620 (16)	0.0435 (11)	−0.0075 (11)	0.0283 (10)	−0.0055 (11)
C15	0.0499 (12)	0.0560 (15)	0.0490 (12)	−0.0044 (11)	0.0326 (11)	−0.0024 (11)
C16	0.0563 (14)	0.0564 (15)	0.0499 (12)	−0.0076 (12)	0.0356 (11)	−0.0034 (11)
C17	0.0561 (14)	0.0660 (17)	0.0474 (12)	−0.0059 (12)	0.0343 (12)	−0.0012 (12)
C18	0.0636 (17)	0.0830 (19)	0.0585 (15)	−0.0028 (15)	0.0371 (14)	−0.0002 (14)
C19	0.0594 (17)	0.108 (3)	0.0619 (16)	−0.0105 (17)	0.0333 (14)	−0.0050 (16)
C20	0.0683 (19)	0.111 (3)	0.0595 (16)	−0.0346 (19)	0.0369 (15)	−0.0147 (17)
C21	0.087 (2)	0.079 (2)	0.0657 (17)	−0.0253 (17)	0.0517 (16)	−0.0161 (14)
C22	0.0625 (15)	0.0657 (18)	0.0586 (14)	−0.0086 (14)	0.0420 (13)	−0.0053 (13)
C23	0.0639 (16)	0.0690 (18)	0.0574 (14)	−0.0034 (14)	0.0400 (13)	−0.0035 (13)
C24	0.0736 (18)	0.089 (2)	0.0699 (17)	0.0089 (17)	0.0474 (16)	−0.0057 (15)
C25	0.0657 (18)	0.121 (3)	0.0718 (18)	0.0061 (18)	0.0463 (16)	−0.0052 (18)
C26	0.0598 (17)	0.125 (3)	0.0759 (18)	−0.0160 (18)	0.0457 (15)	−0.0142 (18)
C27	0.0675 (17)	0.089 (2)	0.0688 (16)	−0.0162 (15)	0.0479 (14)	−0.0145 (14)
C28	0.0586 (15)	0.0649 (17)	0.0520 (13)	−0.0072 (13)	0.0382 (12)	−0.0052 (12)
C29	0.0640 (15)	0.0623 (17)	0.0534 (14)	−0.0100 (13)	0.0392 (12)	−0.0027 (12)
C30	0.0539 (14)	0.0546 (16)	0.0584 (14)	−0.0032 (12)	0.0361 (12)	−0.0080 (12)
C31	0.0794 (18)	0.0630 (18)	0.0679 (16)	−0.0004 (14)	0.0446 (15)	−0.0034 (14)
C32	0.093 (2)	0.0632 (19)	0.095 (2)	−0.0005 (16)	0.0558 (18)	−0.0113 (17)
C33	0.0748 (19)	0.075 (2)	0.081 (2)	−0.0019 (16)	0.0397 (17)	−0.0241 (17)
C34	0.0698 (17)	0.081 (2)	0.0609 (16)	0.0058 (16)	0.0352 (14)	−0.0108 (15)
C35	0.0648 (15)	0.0661 (17)	0.0597 (15)	−0.0032 (13)	0.0387 (13)	−0.0061 (13)

### Geometric parameters (Å, °)

**Table:**

O1—C22	1.384 (4)	C23—C28	1.384 (4)
O1—C23	1.379 (4)	C24—C25	1.369 (5)
O2—C29	1.213 (3)	C25—C26	1.377 (6)
N1—C15	1.472 (3)	C26—C27	1.375 (6)
N1—C29	1.373 (3)	C27—C28	1.390 (4)
N1—C30	1.412 (3)	C30—C31	1.375 (4)
C1—C2	1.428 (4)	C30—C35	1.379 (3)
C1—C6	1.442 (4)	C31—C32	1.386 (4)
C1—C14	1.417 (4)	C32—C33	1.376 (4)
C2—C3	1.359 (5)	C33—C34	1.362 (4)
C3—C4	1.407 (6)	C34—C35	1.392 (4)
C4—C5	1.338 (5)	C2—H2	0.9300
C5—C6	1.422 (6)	C3—H3	0.9300
C6—C7	1.380 (4)	C4—H4	0.9300
C7—C8	1.384 (5)	C5—H5	0.9300
C8—C9	1.424 (4)	C7—H7	0.9300
C8—C13	1.429 (4)	C9—H9	0.9300
C9—C10	1.344 (6)	C10—H10	0.9300
C10—C11	1.396 (5)	C11—H11	0.9300
C11—C12	1.351 (4)	C12—H12	0.9300
C12—C13	1.427 (4)	C15—H15	0.9800
C13—C14	1.426 (3)	C18—H18	0.9300
C14—C15	1.510 (4)	C19—H19	0.9300
C15—C16	1.612 (3)	C20—H20	0.9300
C16—C17	1.502 (3)	C21—H21	0.9300
C16—C28	1.508 (4)	C24—H24	0.9300
C16—C29	1.536 (3)	C25—H25	0.9300
C17—C18	1.388 (4)	C26—H26	0.9300
C17—C22	1.376 (4)	C27—H27	0.9300
C18—C19	1.376 (4)	C31—H31	0.9300
C19—C20	1.368 (6)	C32—H32	0.9300
C20—C21	1.373 (6)	C33—H33	0.9300
C21—C22	1.383 (4)	C34—H34	0.9300
C23—C24	1.381 (5)	C35—H35	0.9300
			
O2···C27	3.410 (4)	C29···H31	2.7700
O2···C31	3.092 (3)	C29···H2	2.9500
O2···H31	2.4800	C30···H2	2.6600
O2···H25^i^	2.8200	C30···H11^iv^	2.8400
O2···H20^ii^	2.8700	C31···H11^iv^	3.0300
N1···C2	2.964 (4)	C33···H19^iii^	2.9200
N1···H2	2.2800	C33···H11^iv^	3.0900
C1···C18	3.354 (3)	C34···H20^iii^	2.8900
C2···C30	3.363 (4)	C34···H11^iv^	2.9300
C2···N1	2.964 (4)	C35···H15	3.0100
C2···C18	3.312 (4)	C35···H11^iv^	2.8000
C3···C21^iii^	3.212 (4)	H2···N1	2.2800
C4···C21^iii^	3.569 (5)	H2···C15	2.8300
C11···C30^iv^	3.584 (4)	H2···C29	2.9500
C12···C16	3.481 (3)	H2···C30	2.6600
C13···C17	3.594 (3)	H2···H18	2.4400
C14···C35	3.496 (3)	H3···C21^iii^	2.9500
C14···C18	3.308 (3)	H5···H7	2.4100
C16···C12	3.481 (3)	H5···H25^vii^	2.5900
C17···C20^ii^	3.579 (4)	H7···H5	2.4100
C17···C13	3.594 (3)	H7···H9	2.4500
C18···C20^ii^	3.470 (5)	H9···H7	2.4500
C18···C2	3.312 (4)	H9···C21^viii^	2.9600
C18···C1	3.354 (3)	H9···H21^viii^	2.4800
C18···C21^ii^	3.547 (4)	H11···C30^iv^	2.8400
C18···C14	3.308 (3)	H11···C31^iv^	3.0300
C19···C22^ii^	3.546 (4)	H11···C33^iv^	3.0900
C20···C17^ii^	3.579 (4)	H11···C34^iv^	2.9300
C20···C18^ii^	3.470 (5)	H11···C35^iv^	2.8000
C21···C18^ii^	3.547 (4)	H12···C15	2.5100
C21···C3^v^	3.212 (4)	H12···C16	2.9300
C21···C4^v^	3.569 (5)	H12···C23	2.9400
C22···C19^ii^	3.546 (4)	H12···C28	2.8000
C27···O2	3.410 (4)	H12···H15	2.0600
C30···C2	3.363 (4)	H15···C12	2.5500
C30···C11^iv^	3.584 (4)	H15···C27	2.9100
C31···O2	3.092 (3)	H15···C35	3.0100
C35···C14	3.496 (3)	H15···H12	2.0600
C1···H18	3.1000	H18···C1	3.1000
C2···H18	2.6900	H18···C2	2.6900
C3···H21^iii^	2.9700	H18···C29	2.6800
C4···H21^iii^	3.0100	H18···H2	2.4400
C12···H15	2.5500	H19···C33^v^	2.9200
C14···H35	2.9000	H20···C34^v^	2.8900
C15···H12	2.5100	H20···O2^ii^	2.8700
C15···H2	2.8300	H21···C3^v^	2.9700
C15···H35	2.7100	H21···C4^v^	3.0100
C16···H12	2.9300	H21···H9^vi^	2.4800
C21···H3^v^	2.9500	H25···H5^ix^	2.5900
C21···H9^vi^	2.9600	H25···O2^x^	2.8200
C23···H12	2.9400	H27···C29	2.6200
C24···H34^iv^	3.0000	H31···O2	2.4800
C25···H35^iv^	3.0900	H31···C29	2.7700
C27···H15	2.9100	H34···C24^iv^	3.0000
C28···H12	2.8000	H35···C14	2.9000
C29···H27	2.6200	H35···C15	2.7100
C29···H18	2.6800	H35···C25^iv^	3.0900
			
C22—O1—C23	117.8 (2)	O2—C29—C16	135.4 (2)
C15—N1—C29	95.39 (18)	N1—C29—C16	93.69 (19)
C15—N1—C30	129.15 (18)	N1—C30—C31	120.3 (2)
C29—N1—C30	132.4 (2)	N1—C30—C35	119.1 (2)
C2—C1—C6	116.1 (3)	C31—C30—C35	120.5 (2)
C2—C1—C14	125.1 (3)	C30—C31—C32	119.7 (3)
C6—C1—C14	118.9 (2)	C31—C32—C33	120.0 (3)
C1—C2—C3	121.7 (3)	C32—C33—C34	120.2 (3)
C2—C3—C4	121.2 (3)	C33—C34—C35	120.6 (3)
C3—C4—C5	119.7 (4)	C30—C35—C34	119.0 (2)
C4—C5—C6	121.6 (4)	C1—C2—H2	119.00
C1—C6—C5	119.6 (3)	C3—C2—H2	119.00
C1—C6—C7	119.8 (3)	C2—C3—H3	119.00
C5—C6—C7	120.6 (3)	C4—C3—H3	119.00
C6—C7—C8	122.6 (3)	C3—C4—H4	120.00
C7—C8—C9	121.8 (3)	C5—C4—H4	120.00
C7—C8—C13	119.0 (2)	C4—C5—H5	119.00
C9—C8—C13	119.2 (3)	C6—C5—H5	119.00
C8—C9—C10	121.7 (3)	C6—C7—H7	119.00
C9—C10—C11	119.5 (3)	C8—C7—H7	119.00
C10—C11—C12	121.2 (3)	C8—C9—H9	119.00
C11—C12—C13	122.0 (3)	C10—C9—H9	119.00
C8—C13—C12	116.3 (2)	C9—C10—H10	120.00
C8—C13—C14	120.0 (3)	C11—C10—H10	120.00
C12—C13—C14	123.7 (3)	C10—C11—H11	119.00
C1—C14—C13	119.7 (3)	C12—C11—H11	119.00
C1—C14—C15	125.8 (2)	C11—C12—H12	119.00
C13—C14—C15	114.6 (2)	C13—C12—H12	119.00
N1—C15—C14	121.8 (2)	N1—C15—H15	109.00
N1—C15—C16	87.01 (16)	C14—C15—H15	109.00
C14—C15—C16	119.02 (19)	C16—C15—H15	109.00
C15—C16—C17	116.0 (2)	C17—C18—H18	119.00
C15—C16—C28	114.08 (19)	C19—C18—H18	119.00
C15—C16—C29	83.91 (17)	C18—C19—H19	120.00
C17—C16—C28	111.2 (2)	C20—C19—H19	120.00
C17—C16—C29	116.5 (2)	C19—C20—H20	120.00
C28—C16—C29	112.8 (2)	C21—C20—H20	120.00
C16—C17—C18	122.2 (2)	C20—C21—H21	120.00
C16—C17—C22	120.2 (3)	C22—C21—H21	120.00
C18—C17—C22	117.6 (2)	C23—C24—H24	120.00
C17—C18—C19	121.3 (3)	C25—C24—H24	120.00
C18—C19—C20	119.8 (3)	C24—C25—H25	120.00
C19—C20—C21	120.4 (3)	C26—C25—H25	120.00
C20—C21—C22	119.3 (3)	C25—C26—H26	120.00
O1—C22—C17	122.8 (2)	C27—C26—H26	120.00
O1—C22—C21	115.6 (3)	C26—C27—H27	119.00
C17—C22—C21	121.6 (3)	C28—C27—H27	119.00
O1—C23—C24	115.8 (2)	C30—C31—H31	120.00
O1—C23—C28	122.5 (3)	C32—C31—H31	120.00
C24—C23—C28	121.7 (3)	C31—C32—H32	120.00
C23—C24—C25	119.9 (3)	C33—C32—H32	120.00
C24—C25—C26	119.7 (4)	C32—C33—H33	120.00
C25—C26—C27	120.2 (4)	C34—C33—H33	120.00
C26—C27—C28	121.4 (3)	C33—C34—H34	120.00
C16—C28—C23	120.2 (3)	C35—C34—H34	120.00
C16—C28—C27	122.7 (2)	C30—C35—H35	120.00
C23—C28—C27	117.1 (3)	C34—C35—H35	121.00
O2—C29—N1	130.9 (2)		
			
C23—O1—C22—C21	164.1 (2)	N1—C15—C16—C29	0.4 (2)
C22—O1—C23—C24	−163.8 (2)	C14—C15—C16—C17	8.8 (3)
C22—O1—C23—C28	16.9 (3)	N1—C15—C16—C17	−116.2 (2)
C23—O1—C22—C17	−14.8 (4)	N1—C15—C16—C28	112.6 (2)
C29—N1—C30—C31	10.4 (5)	C14—C15—C16—C29	125.4 (2)
C29—N1—C30—C35	−167.0 (3)	C14—C15—C16—C28	−122.3 (2)
C29—N1—C15—C16	−0.5 (2)	C29—C16—C28—C27	29.6 (3)
C15—N1—C30—C35	−12.0 (5)	C29—C16—C28—C23	−152.6 (2)
C30—N1—C15—C14	75.2 (4)	C15—C16—C28—C27	−63.9 (3)
C15—N1—C29—C16	0.5 (2)	C15—C16—C29—O2	177.0 (4)
C30—N1—C15—C16	−162.2 (3)	C15—C16—C28—C23	113.9 (2)
C29—N1—C15—C14	−123.1 (2)	C28—C16—C17—C18	−160.8 (2)
C30—N1—C29—C16	161.3 (3)	C28—C16—C17—C22	21.6 (3)
C15—N1—C30—C31	165.4 (3)	C29—C16—C17—C18	−29.7 (4)
C15—N1—C29—O2	−177.1 (4)	C29—C16—C17—C22	152.7 (3)
C30—N1—C29—O2	−16.3 (6)	C15—C16—C17—C18	66.7 (3)
C2—C1—C14—C13	−175.2 (2)	C17—C16—C28—C23	−19.6 (3)
C2—C1—C6—C7	179.2 (2)	C17—C16—C28—C27	162.6 (2)
C6—C1—C14—C15	−178.4 (2)	C15—C16—C29—N1	−0.4 (2)
C14—C1—C6—C5	−178.1 (2)	C15—C16—C17—C22	−110.9 (3)
C2—C1—C6—C5	0.6 (3)	C28—C16—C29—N1	−114.0 (2)
C14—C1—C6—C7	0.5 (3)	C28—C16—C29—O2	63.4 (4)
C14—C1—C2—C3	175.7 (2)	C17—C16—C29—O2	−66.9 (5)
C6—C1—C14—C13	3.4 (3)	C17—C16—C29—N1	115.7 (3)
C6—C1—C2—C3	−2.9 (3)	C16—C17—C22—C21	175.7 (3)
C2—C1—C14—C15	3.1 (3)	C22—C17—C18—C19	2.0 (4)
C1—C2—C3—C4	2.6 (4)	C18—C17—C22—O1	176.8 (2)
C2—C3—C4—C5	0.2 (4)	C16—C17—C18—C19	−175.6 (3)
C3—C4—C5—C6	−2.5 (5)	C18—C17—C22—C21	−2.0 (4)
C4—C5—C6—C1	2.0 (4)	C16—C17—C22—O1	−5.5 (4)
C4—C5—C6—C7	−176.6 (3)	C17—C18—C19—C20	−0.8 (5)
C1—C6—C7—C8	−2.6 (4)	C18—C19—C20—C21	−0.6 (5)
C5—C6—C7—C8	176.0 (3)	C19—C20—C21—C22	0.6 (5)
C6—C7—C8—C13	0.7 (4)	C20—C21—C22—C17	0.8 (5)
C6—C7—C8—C9	−178.6 (2)	C20—C21—C22—O1	−178.2 (3)
C7—C8—C13—C14	3.3 (3)	O1—C23—C24—C25	−178.4 (3)
C7—C8—C9—C10	177.0 (3)	C28—C23—C24—C25	1.0 (4)
C13—C8—C9—C10	−2.3 (4)	O1—C23—C28—C16	1.3 (4)
C9—C8—C13—C12	4.0 (3)	C24—C23—C28—C16	−178.1 (2)
C7—C8—C13—C12	−175.3 (2)	C24—C23—C28—C27	−0.1 (4)
C9—C8—C13—C14	−177.4 (2)	O1—C23—C28—C27	179.2 (2)
C8—C9—C10—C11	−1.2 (4)	C23—C24—C25—C26	−1.2 (5)
C9—C10—C11—C12	2.9 (4)	C24—C25—C26—C27	0.5 (5)
C10—C11—C12—C13	−1.0 (4)	C25—C26—C27—C28	0.3 (4)
C11—C12—C13—C8	−2.4 (3)	C26—C27—C28—C16	177.4 (2)
C11—C12—C13—C14	179.0 (2)	C26—C27—C28—C23	−0.6 (4)
C12—C13—C14—C15	−5.3 (3)	N1—C30—C31—C32	−175.2 (3)
C8—C13—C14—C15	176.22 (19)	C35—C30—C31—C32	2.1 (5)
C12—C13—C14—C1	173.2 (2)	N1—C30—C35—C34	176.1 (3)
C8—C13—C14—C1	−5.4 (3)	C31—C30—C35—C34	−1.3 (5)
C1—C14—C15—N1	14.0 (3)	C30—C31—C32—C33	−1.3 (6)
C1—C14—C15—C16	−91.9 (3)	C31—C32—C33—C34	−0.4 (6)
C13—C14—C15—C16	86.4 (2)	C32—C33—C34—C35	1.2 (5)
C13—C14—C15—N1	−167.69 (18)	C33—C34—C35—C30	−0.4 (5)

Symmetry codes: (i) −*x*+1, *y*+1/2, −*z*+1/2; (ii) −*x*, −*y*+1, −*z*; (iii) −*x*, *y*+1/2, −*z*+1/2; (iv) −*x*+1, −*y*+1, −*z*+1; (v) −*x*, *y*−1/2, −*z*+1/2; (vi) *x*, −*y*+1/2, *z*−1/2; (vii) *x*−1, *y*, *z*; (viii) *x*, −*y*+1/2, *z*+1/2; (ix) *x*+1, *y*, *z*; (x) −*x*+1, *y*−1/2, −*z*+1/2.

### Hydrogen-bond geometry (Å, °)

**Table:**

*D*—H···*A*	*D*—H	H···*A*	*D*···*A*	*D*—H···*A*
C2—H2···N1	0.93	2.28	2.964 (4)	130
C31—H31···O2	0.93	2.48	3.092 (3)	124
C3—H3···Cg1^iii^	0.93	2.86	3.601 (3)	138
C11—H11···Cg2^iv^	0.93	2.63	3.543 (3)	166

Symmetry codes: (iii) −*x*, *y*+1/2, −*z*+1/2; (iv) −*x*+1, −*y*+1, −*z*+1.
